# Early functional deficits following first‐time acute lateral ankle sprain: A multidimensional assessment framework with clinically relevant cut‐off values

**DOI:** 10.1002/jeo2.70892

**Published:** 2026-08-27

**Authors:** Janina Tennler, Christian Raeder, Arthur Praetorius, Sebastian Colcuc, Eva S. Steinhausen, Marcel Dudda, Christian Schoepp

**Affiliations:** ^1^ Department of Arthroscopic Surgery, Sports Traumatology and Sports Medicine BG Klinikum Duisburg Duisburg Germany; ^2^ Athletikum Rhein Ruhr BG Klinikum Duisburg Duisburg Germany; ^3^ Department of Trauma, Hand and Reconstructive Surgery University Hospital Essen Essen Germany; ^4^ Department of Trauma and Reconstructive Surgery BG Klinikum Duisburg Duisburg Germany

**Keywords:** clinical decision‐making, gait analysis, lateral ankle sprain, muscle strength testing, sensorimotor function

## Abstract

**Purpose:**

Lateral ankle sprain (LAS) is one of the most common musculoskeletal injuries associated with sensorimotor impairments that increase the risk of reinjury and chronic ankle instability. Early clinical assessment after acute LAS is often symptom‐guided rather than based on objective functional testing. This study aimed to identify the most sensitive functional impairments within the first weeks after injury and to determine cut‐off values that support structured clinical assessment.

**Methods:**

In this cross‐sectional matched case‐control analysis, 36 individuals (14–41 years) with MRI‐confirmed first‐time acute LAS were compared to 36 matched healthy controls. Patients (recruiting 01/22‐09/24) were assessed 2–3 weeks post‐injury using patient‐reported outcomes, postural control, strength, range of motion (ROM), gait/running kinematics and jump performance. Between‐group differences were analysed using *t* tests or Statistical Parametric Mapping. Receiver operating characteristic analyses were used to determine diagnostic cut‐off values. It was hypothesized that individuals with acute first‐time LAS would demonstrate clinically relevant deficits across functional domains compared with healthy controls, and that these parameters would allow the derivation of clinically useful cut‐off values.

**Results:**

Individuals with LAS demonstrated substantial impairments across several domains. The most discriminative parameters were Foot and Ankle Ability Measure (FAAM) Sport (effect size [ES] −4.09, cut‐off <86) and Cumberland Ankle Instability Tool (CAIT) (ES −3.65; cut‐off <26) (psychometry), Star Excursion Balance Test (SEBT) composite score (ES −1.22; cut‐off <92.81) (postural control), concentric plantarflexion strength (ES −1.34; cut‐off <2.24 Nm/kg) (strength), passive plantarflexion and total ROM (ES −2.29; cut‐off <44° and <72°), transverse ankle kinematics during gait (ES 1.14; cut‐off <−15°) (gait/running) and drop jump contact time (ES 1.62; cut‐off >0.23 s) (jump performance). These measures formed a minimal multidimensional assessment.

**Conclusion:**

These parameters and cut‐off values enable objective, multidimensional assessment in the early phase after acute LAS and may facilitate impairment‐based rehabilitation beyond symptom‐guided evaluation.

**Level of Evidence:**

Level III, cross‐sectional matched pair‐study.

## Graphical abstract

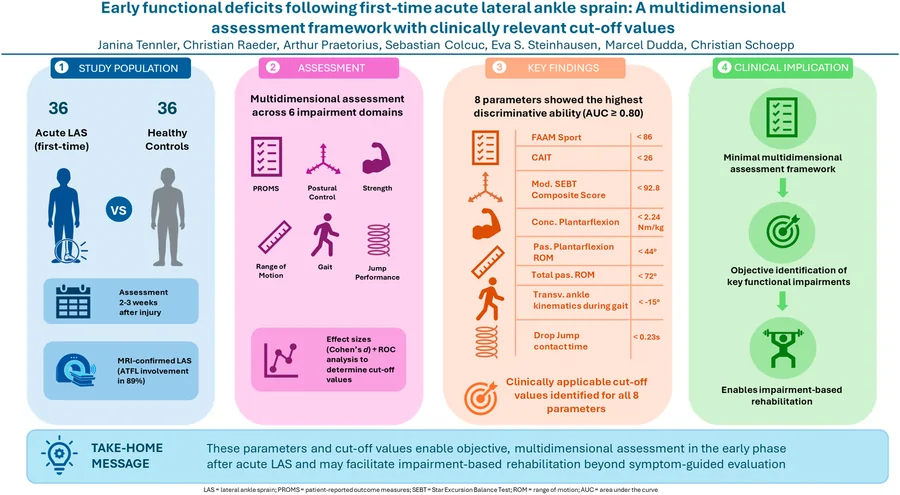

**Abbreviations**
BMI: body mass index
CAIT: Cumberland Ankle Instability Tool
CI: confidence interval
DJ: drop jump
FAAM: Foot and Ankle Ability Measure
LAS: lateral ankle sprain
MCID: minimal clinically important differences
PF: plantarflexion
RCT: randomized controlled trial
ROAST: Rehabilitation‐Oriented‐Assessment
ROC: receiver operating characteristic
ROM: range of motion
SEBT: Star Excursion Balance Test
SEM: standard error of measurement
SPM: Statistical Parametric Mapping
TTB: time‐to‐boundary
VGRF: vertical ground reaction force

## INTRODUCTION

The lateral ankle sprain (LAS) is among the most common injuries in sports and general population, with incidence rates of 5–7 sprains per 1000 person‐years [4, 12, 14, 16, 31]. LAS is associated with substantial mechanical and sensorimotor impairments that may contribute to recurrent sprains and chronic ankle instability (CAI) [6, 19]. Among all lower limb musculoskeletal injuries, LAS exhibits the highest reinjury rate [6], with a twofold increased risk within the first year [33]. These findings highlight that inadequate management in the acute phase may have lasting functional implications.

Early identification of functional impairments following acute LAS is considered essential for guiding rehabilitation and preventing recurrent injury. However, routine clinical assessment in the early rehabilitation phase remains challenging since decision‐making is often primarily based on symptom resolution and general clinical examination rather than on structured functional assessment [6]. As a result, relevant deficits in sensorimotor function, strength or dynamic stability remain undetected during early rehabilitation.

Recent consensus recommendations have emphasized the importance of early multidimensional assessment following LAS. The Rehabilitation‐Oriented Assessment (ROAST) framework proposed by the International Ankle Consortium highlights impairment domains that should be considered after acute LAS. These include patient‐reported outcomes, postural control, strength, range of motion (ROM) and functional performance. However, their application is limited because they rely on subjective clinician ratings or insufficient assessments. The PAASS framework, developed to support return‐to‐sport decision‐making after acute ankle sprain, recommends consideration of pain, ankle impairments, athlete perception, sensorimotor control and sport/functional performance. However, these recommendations primarily target the later stages of rehabilitation and do not provide an objective threshold to guide clinical assessment during the early post‐injury phase [29]. In addition, the recently proposed Ankle‐GO test battery provides a structured return‐to‐sport assessment including functional performance tests and decision thresholds [25]. However, objective cut‐off values for the identification of clinically relevant impairments during the early phase following a first‐time LAS remain scarce. As a result, clinicians often lack objective criteria to determine the presence, magnitude and clinical relevance of functional impairments in the early phase after acute injury [6].

Therefore, the aim of this study was to identify the most sensitive functional parameters that can discriminate between individuals with acute LAS and healthy controls across multiple impairment domains. Furthermore, clinically applicable cut‐off values were determined to support structured clinical assessment. It was hypothesized that individuals with first‐time acute LAS would demonstrate clinically relevant impairments across functional domains compared with healthy controls and that these parameters would allow the derivation of clinically useful cut‐off values.

## METHODS

### Study design

**Figure 1:**
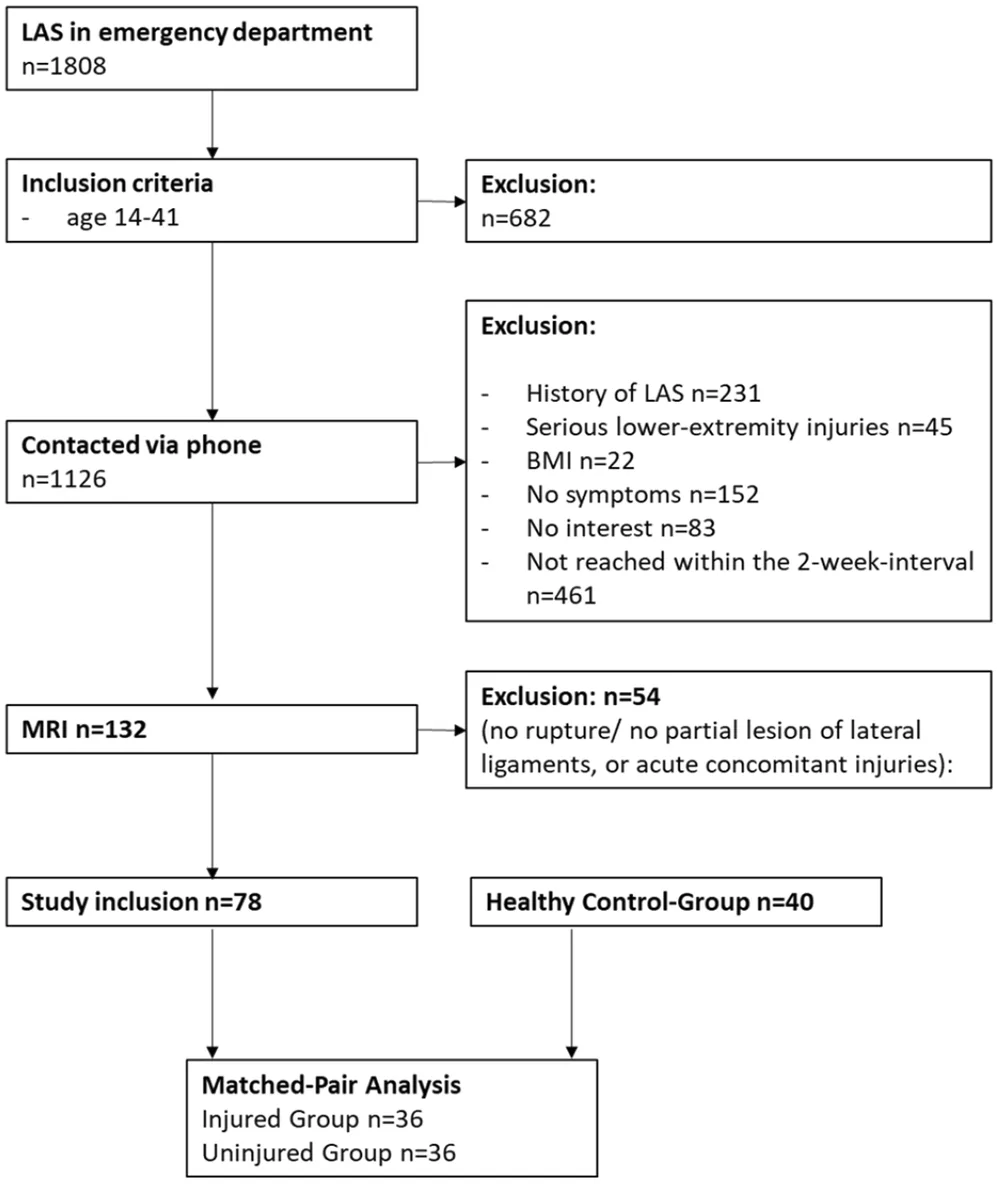
Consort flow diagram. BMI, body mass index; LAS, lateral ankle sprain; MRI, magnetic resonance imaging.

This cross‐sectional analysis is part of a larger randomized controlled trial (RCT) (OSGAR‐study [30]) and could be described as a convenience sample, which was prospectively registered in the International Standard Randomised Controlled Trial Number (ISRCTN) registry (13640422) and the German Clinical Trials Registry (DRKS (DRKS00026049). The sample size (*n* = 78 injured participants; *n* = 40 uninjured participants) was determined by the larger RCT (Figure 1). A post hoc matched case‐control analysis was conducted of the INJURED and UNINJURED groups with fixed (sex, smoking and sport intensity) and variable matching criteria (age and body mass index [BMI]) to maximize comparability between groups and reduce potential confounding (Figure 1 and Table 1). A healthy control group was selected instead of the contralateral limb, as previous studies have reported bilateral sensorimotor adaptations following unilateral ankle sprain [7, 8, 11, 22].

**Table 1:** Demographic information for sex, age, height, weight, BMI, Tegner Score and MRI characteristics.

Injured *n* = 36		Uninjured *n* = 36	*p* Value
Sex			
Male: *n* = 22 (61%)		Male: *n* = 22 (61%)	—
Female: *n* = 14 (39%)		Female: *n* = 14 (39%)	
Age (years)			
22.1 ± 6.7		24.7 ± 6.2	0.088
Height (m)			
1.8 ± 0.1		1.8 ± 0.1	0.489
Weight (kg)			
72.6 ± 12.9		70.7 ± 10.9	0.513
BMI			
23.0 ± 2.5		22.1 ± 2.5	0.138
Tegner Score			
6 ± 3		6 ± 2	0.07
MRI characteristics			
Single‐ligament injury	*n* = 12 (33.3%)		
Two‐ligament injury	*n* = 24 (66.7%)		
ATFL involved	*n* = 32 (88.9%)		
CFL involved	*n* = 24 (66.7%)		
PTFL involved	*n* = 4 (11.1%)		

*Note*: Comparison of group means (mean ± SD) for age, height, weight, BMI and Tegner Score was determined using an unpaired *t* test.

Abbreviations: ATFL, anterior talofibular ligament; BMI, body mass index; CFL, calcaneofibular ligament; MRI, magnetic resonance imaging; PTFL, posterior talofibular ligament; SD, standard deviation.

All participants provided written informed consent before data collection. Ethical approval was granted by the Institutional Review Board of the Ärztekammer Nordrhein (ref: 2021236 28/09/2021). The detailed study protocol of the study has already been published in Tennler et al. [30].

Inclusion criteria (as part of the larger RCT) were ICD‐Code S93.4x (‘sprain of the ankle’), age 14–41 years, BMI 19–30 and an MRI‐confirmed rupture of at least one lateral ankle ligament (recruited from January 2022 to September 2024). Participants with acute concomitant injuries of the ankle, pre‐injuries of the injured and non‐injured ankle, serious lower‐extremity injuries of the last 6 months or neurological diseases or impairments of the vestibular system were excluded. The healthy control group had no history of ankle injuries.

### Protocol

Diagnostic testing within a 2–3‐week post‐injury window covered several impairment domains after LAS [6, 23]. Following diagnosis in the emergency department, all patients received standardized initial conservative management consisting of functional ankle bracing, analgesic medication as required and pain‐adapted partial weight‐bearing before participation in the study. Given the rapid recovery processes occurring during this period, some heterogeneity in functional status cannot be excluded.

#### Psychometrics

The Cumberland Ankle Instability Tool (CAIT) questionnaire was used to measure perceived ankle joint function, as well as the Foot and Ankle Ability Measure (FAAM) questionnaire to assess subjective impairments of ankle function [15, 21].

#### Postural control

Static postural control was measured by the single‐leg stance with eyes open and closed (10 s each) on a pressure plate of a treadmill (h/p/cosmos sports & medical GmbH). During the trial, centre of pressure (COP) parameters (area, velocity, trace) were tracked, and a time‐to‐boundary (TTB) analysis was performed [13]. Trials were invalid if the participants removed their hands from their hips, touched the ground with their non‐stance limb or lifted either the forefoot or heel of the stance limb.

Dynamic postural control was measured via the mod. Star Excursion Balance Test (SEBT). Anterior, posteromedial and posterolateral reach distance of the swing leg (cm) was measured, and the composite score was calculated [26].

#### Strength

Bilateral force production (N) in a closed kinetic chain measurement, as well as plantarflexion (PF) and dorsiflexion torque (Nm) in an open kinetic chain, was tested in a motorized dynamometer (IsoMed, D. & R. Ferstl GmbH). Eversion strength was isometrically tested supine with full knee joint extension using a handheld dynamometer (Lafayette, model 01165A).

#### Range of motion

Sagittal ankle ROM was quantified using the angular output of the motorized dynamometer, which provided an objective recording of the ankle joint angle, while lying in a supine position. The ankle was both moved passively and actively into maximal PF (+°) and dorsiflexion (−°).

#### Proprioception

Joint repositioning sense of PF and dorsiflexion was tested according to Konradsen and Magnusson [18]. The participant had the task of actively reproducing a reference angle. Constant error, variable error and absolute error were calculated [28].

#### Gait and running

The gait and running analysis was performed at 5 and 10 km/h (30 s each) on a motorized treadmill (h/p/cosmos sports & medical GmbH) with an integrated pressure plate (FDM‐THQ 2i, Zebris Medical GmbH) sampling at 300 Hz.

#### Jump‐performance and landing‐strategy

A single‐leg drop landing and a bilateral drop jump (DJ), each from a 20 cm box onto two parallel independent force plates (FP‐4060–08; Bertec Corporation) to measure vertical ground reaction force (VGRF), were conducted. Participants were instructed to minimize ground contact time and maximize rebound height during the DJ. Performance parameters like jump height, contact time and the resulting reactive strength index were calculated. During the single‐leg drop landing, the participant had to step forward and fall onto the force plate, land on a single leg and maintain that position.

Prior to jump and landing assessments, participants were progressively familiarized with the testing procedures. Jump tasks were only performed if participants reported no pain and felt confident completing the task. Participants could refuse or discontinue testing at any time. No adverse events occurred during testing. Assessments were performed without bracing or taping to avoid influencing biomechanical outcomes.

### Data processing

Kinematic data were collected at 200 Hz using a portable motion capture system (myoMotion, Noraxon U.S.A. Inc.). A total of seven inertial measurement unit (IMU) sensors were placed on the pelvis, both femurs, tibiae and feet. Standard error of measurement (SEM) values were used to assess whether the measured differences fall within the expected measurement error range [2].

### Data analysis and statistical approach

For each UNINJURED participant, one limb was assigned as involved, so that an equal proportion of right and left limbs was classified as involved in both groups [9, 10]. For some variables, analyses were conducted using the available data (complete‐case analysis) as some patients were unable to perform specific tests. No imputation was performed.

All variables were analysed without categorization. Analyses focused on between‐group differences. No subgroup or interaction analyses were conducted. As participants were recruited from predefined groups using a convenience sample, no adjustment for sampling strategy was required. Discrete outcome parameters were analysed using an unpaired *t* test using JASP (JASP, 2018). In case of violation of normality, the Mann–Whitney *U* test was performed. Associated effect sizes were calculated (Cohen's *d*).

Statistical Parametric Mapping (SPM) *t* tests were used to statistically compare continuous data [24]. In case normality was violated, a corresponding non‐parametric SPM test was used (SnPM). The open‐source spm1d package (v. 0.4, www.spm1d.org, Pataky, 2012) was used in MATLAB (R2022b, The MathWorks Inc).

To determine the optimal cut‐off value for differentiating between the two groups, a logistic regression, receiver operating characteristic (ROC) curves and the Youden Index were performed [32]. Analysis was carried out with a self‐written MATLAB script (R2022b, The MathWorks Inc). To identify the most clinically relevant assessment parameters, significant variables were ranked according to their Cohen's *d* effect size; the variable with the highest effect size was selected.

Given the exploratory objective of identifying potentially relevant assessment parameters across multiple functional domains, the interpretation of the results was based not only on statistical significance but also on effect sizes, clinical relevance (including minimal clinically important differences [MCIDs] where available) and diagnostic performance.

Pearson's correlation coefficient (*r*) was applied to analyse the relationships between the parameters of the assessment framework. In cases where normality was not met, Spearman's rank correlation coefficient (*p*) was applied. The strength of the correlation was interpreted based on Cohen's guidelines.

Statistical analysis and presentation of this study are consistent with the Checklist for Statistical Assessment of Medical Papers (CHAMP) statement [20].

## RESULTS

Following the predefined matching procedure, 36 individuals with first‐time acute LAS and 36 matched healthy controls were included in the final analysis (Figure 1, Table 1).

MRI findings revealed predominantly combined ligament injuries, with 24 of 36 participants (66.7%) presenting a two‐ligament injury and 12 (33.3%) an isolated ligament injury. The anterior talofibular ligament was involved in 88.9% of participants, followed by the calcaneofibular ligament (66.7%) and posterior talofibular ligament (11.1%) (Table 1).

Because of the large number of outcomes, detailed statistical results (including means, standard deviations, effect sizes and *p* values) are provided in Data S1. The main text focuses on the parameters included in the final multidimensional assessment framework. All estimates represent unadjusted between‐group comparisons. No confounder adjustment was performed.

### Psychometrics

CAIT scores were significantly lower in the INJURED group compared to the UNINJURED group (*p *< 0.001, large effect size) (Table 2). Group differences exceeded the published MCID (≥3 points) [34].

**Table 2:** Comparison of group means for psychometric measures (CAIT and FAAM).

	Injured *n* = 36	Uninjured *n* = 36	*p* Value	Cohen's *d*
CAIT	13.4 ± 5.6	28.8 ± 2.1	<0.001***^†^	−3.652
FAAM ADL	65.4 ± 23.3	99.8 ± 0.7	<0.001***^†^	−2.114
FAAM Sport	38.0 ± 21.7	99.3 ± 2.4	<0.001***^†^	−4.085
FAAM Total	56.5 ± 21.9	99.6 ± 1.4	<0.001***^†^	−2.887

*Note*: Significance was determined using an unpaired *t* test (^†^). Effect sizes were calculated using Cohen's *d*. Significance levels: ***p < 0.001.

Abbreviations: ADL, activities of daily living; CAIT, Cumberland Ankle Instability Tool; FAAM, Foot and Ankle Ability Measure.

All FAAM subscales (activities of daily living [ADL], Sport, Total) differed significantly (*p *< 0.001, large effect sizes) (Table 2). Group differences in ADL and Sport both exceeded the MCID (>8 and >9 points, respectively) [21].

### Postural control

During eyes‐open single‐leg stance, COP parameters (area, trace, velocity) were significantly higher in the INJURED group (*p *< 0.05, medium effect sizes) (Table 3). TTB showed significant group differences in 2/6 parameters under eyes‐open (*p* < 0.05, medium effect sizes) and 1/6 under eyes‐closed conditions (see Data S1).

**Table 3:** Comparison of group means (mean ± SD) for COP parameters.

		Injured *n* = 36	Uninjured *n* = 36		
		(Not feasible *n* = 1)	(Not feasible *n* = 1)	*p* Value	Cohen's *d*
Eyes open	COP area (mm^2^)	449 ± 187	347 ± 145	0.013*^†^	0.609
	COP trace (mm)	337 ± 105	273 ± 94	0.010**^†^	0.631
	COP velocity (mm/s)	34 ± 10	27 ± 9	0.010**^†^	0.632

		**(Not feasible *n*** = **6)**	**(Not feasible *n*** = **1)**		
Eyes closed	COP area (mm^2^)	2397 ± 1023	1985 ± 1040	0.086^†^	0.434
	COP trace (mm)	864 ± 266	751 ± 244	0.063^†^	0.470
	COP velocity (mm/s)	86 ± 26	75 ± 24	0.059^†^	0.478

*Note*: Significance was determined using an unpaired *t* test (^†^). Effect sizes were calculated using Cohen's *d*. Significance levels: *p < 0.05, **p < 0.01.

Abbreviations: COP, centre of pressure; SD, standard deviation.

In the mod. SEBT, all reach directions and the composite score were significantly lower in the INJURED group (*p* < 0.001, large effect sizes) (see Data S1). The composite score was reduced by 13% (89 ± 11 vs. 102 ± 10), exceeding the MCID of 3.5% [5].

### Strength

All strength outcomes were significantly reduced in the INJURED group (Table 4).

**Table 4:** Comparison of group means (mean ± SD) for strength measurements normalized to bodyweight.

	Injured *n* = 36	Uninjured *n* = 36	*p* Value	Cohen's *d*
Leg press (N/kg)				
MVIC	24.35 ± 5.05	27.23 ± 5.74	0.027*^†^	−0.534

	(not feasible *n* = 1)	(not feasible *n* = 1)		
Max concentric	30.68 ± 6.37	36.17 ± 6.05	<0.001***^†^	−0.885
Max eccentric	34.44 ± 7.34	41.05 ± 8.07	<0.001***^†^	−0.857
Dorsiflexion (Nm/kg)				
Max concentric	0.5 ± 0.13	0.56 ± 0.09	0.011*^†^	−0.613
Max eccentric	0.53 ± 0.13	0.61 ± 0.09	0.002**^†^	−0.752
Plantarflexion (Nm/kg)				
Max concentric	1.64 ± 0.84	2.6 ± 0.57	<0.001***^†^	−1.337
Max eccentric	1.89 ± 0.93	2.9 ± 0.7	<0.001***^†^	−1.225
Eversion (N/kg)				
Isometric	1.82 ± 0.68	2.51 ± 0.61	0.001***^†^	−1.070

*Note*: Significance was determined using an unpaired *t* test (^†^). Effect sizes were calculated using Cohen's *d*. Significance levels: *p < 0.05, **p < 0.01, ***p < 0.001.

Abbreviations: MVIC, maximal voluntary isometric contraction; SD, standard deviation.

### Range of motion

Active and passive dorsiflexion, PF and total ankle ROM were significantly reduced in the INJURED group (*p *< 0.001) (Table 5).

**Table 5:** Comparison of group means (mean ± SD) for ROM parameters.

	Injured *n* = 36 (not feasible *n* = 4)	Uninjured *n* = 36 (not feasible *n* = 13)	*p* Value	Cohen's *d*
Passive (°)				
Dorsiflexion	−22 ± 7	−31 ± 5	<0.001***^†^	1.524
Plantarflexion	32 ± 8	48 ± 6	<0.001***^†^	−2.289
ROM	53 ± 16	80 ± 9	<0.001***^†^	−2.289
Active (°)				
Dorsiflexion	−18 ± 5	−22 ± 4	0.008**^†^	0.75
Plantarflexion	28 ± 8	44 ± 7	<0.001***^†^	−2.208
ROM	45 ± 13	66 ± 8	<0.001***^†^	−2.113

*Note*: Significance was determined using an unpaired *t* test (^†^). Effect sizes were calculated using Cohen's *d*. Significance levels: **p < 0.01, ***p < 0.001.

Abbreviations: ROM, range of motion; SD, standard deviation.

### Proprioception

No significant group differences were found for constant, variable or absolute error (see Data S1).

### Gait and running

During gait, participants in the INJURED group showed significantly reduced VGRFs (normalized to body weight) during terminal stance (48%–49% of the gait cycle, *p* < 0.001, *d* = –0.83). In addition, ankle motion differed between groups: the INJURED group demonstrated less internal ankle rotation during pre‐swing (55%–67% of the gait cycle, *p* = 0.002, *d* = 1.14, exceeding SEM [2]). During running, the INJURED group exhibited reduced hip extension during stance and early float (31%–46% of the cycle, *p* = 0.017, *d* = 1.09, exceeding SEM [2]) (see Data S1).

### Jump‐performance and landing‐strategy

DJ performance was reduced in the INJURED group, with significantly lower jump height, longer contact times (0.27 ± 0.06 vs. 0.2 ± 0.04) and reduced RSI (*p* < 0.001, medium–large effects). Maximum total VGRF and VGRF of the involved leg were also significantly lower (*p* < 0.001, large effects) (see Data S1).

During single‐leg drop landings, the INJURED group consistently showed altered loading and joint kinematics (see Data S1):

‐ VGRF: reduced during peak landing (58%–64%, *p* = 0.003, *d* = –1.08).
‐ Ankle kinematics: reduced PF during late flight/early landing (34%–52%, *p* = 0.025, *d* = 0.90, exceeding SEM [2]) and reduced eversion during peak landing (64–67%, *p* = 0.023, *d* = 0.70).
‐ Hip kinematics: increased abduction during flight (14%–36%, *p* = 0.007, *d* = 0.80, exceeding SEM [2]) and reduced internal rotation both in early flight (0%–11%, *p* = 0.009, *d* = 0.76, exceeding SEM [2]) and mid‐flight (31%–36%, *p* = 0.021, *d* = 0.69, exceeding SEM [2]).

### Multidimensional assessment framework

Table 6 presents the significant parameters that distinguish between the INJURED and UNINJURED groups, ranked by effect size. Parameters with the highest effect sizes were identified for each test domain to determine the most clinically relevant parameters for functional assessment after acute LAS. As there were no significant differences in proprioception, this test domain was not included in the final assessment framework. In the psychometric test domain, two questionnaires (FAAM Sport and CAIT) exhibited the highest effect size by a considerable margin. As they assess different aspects—with the FAAM Sport being primarily sports‐related—both were included to ensure a comprehensive evaluation. Table 7 shows the corresponding optimal cut‐off values, along with sensitivity, specificity, Youden Index and AUC.

**Table 6:** Significant parameters with their corresponding Cohen's *d* effect size.

Parameter	Effect size		Parameter	Effect size	
FAAM Sport	−4.09	Large	SLDL hip kinematics (frontal) (20 cm)	0.80	Medium
CAIT	−3.65		SLDL hip kinematics (transverse) (20 cm)	0.76	
ROM passive plantarflexion	−2.29		ROM active dorsiflexion	0.75	
ROM passive	−2.29		Dorsiflexion (eccentric)	−0.75	
FAAM total	−2.89		SLDL ankle kinematics (frontal) (20 cm)	0.70	
ROM active plantarflexion	−2.21		Drop jump RSI (20 cm)	−0.68	
FAAM ADL	−2.11		TTB AP min (eyes open)	−0.67	
ROM active	−2.11		Drop jump height (20 cm)	−0.65	
Drop jump contact time (20 cm)	1.62		COP velocity (eyes open)	0.63	
ROM passive dorsiflexion	1.52		COP trace (eyes open)	0.63	
Drop jump rel. VGRF involved leg (20 cm)	−1.37		COP area (eyes open)	0.61	
Plantarflexion (concentric)	−1.34		Dorsiflexion (concentric)	−0.61	
Drop jump rel. VGRF (20 cm)	−1.24		TTB AP min mean (eyes open)	−0.57	
Plantarflexion (eccentric)	−1.23		TTB AP min mean (eyes closed)	−0.57	
SEBT Composite Score	−1.22		Leg press (isometric)	−0.53	
SEBT posterior‐medial reach	−1.15				
Gait ankle kinematics (transverse)	1.14				
Running hip kinematics (sagittal)	1.09				
SLDL rel. VGRF (20 cm)	−1.08				
Eversion (isometric)	−1.07				
SEBT posterior–lateral reach	−1.01				
SEBT anterior reach	−0.99				
SLDL ankle kinematics (sagittal) (20 cm)	0.90				
Leg press (concentric)	−0.89				
Leg press (eccentric)	−0.86				
Gait VGRF	−0.83				

Abbreviations: ADL, activities of daily living; CAIT, Cumberland Ankle Instability Tool; COP, centre of pressure; FAAM, foot and ankle ability measure; ROM, range of motion; RSI, reactive strength index; SEBT, Star Excursion Balance Test; SLDL, single‐leg drop landing; TTB AP, time‐to‐boundary anterior–posterior; VGRF, vertical ground reaction force.

**Table 7:** Summary of cut‐off value, sensitivity, specificity, Youden index and AUC with 95% confidence intervals for the recommended assessment framework.

Domain	Parameter	Optimal cut‐off	Optimal sensitivity	Optimal specificity	Optimal Youden index	Optimal AUC
Psychometry	CAIT	<26	0.94	1	0.94	0.99 [0.98, 1]
	FAAM sport	<86	1	1	1	1 [1, 1]
Range of motion	ROM passive plantarflexion	<44°	0.83	0.97	0.79	0.95 [0.89, 0.99]
	Total passive ROM	<72°	0.83	0.94	0.76	0.95 [0.89, 0.99]
Gait/Running	External ankle rotation (54%–66% gait cycle)	<−15°	0.74	0.79	0.53	0.8 [0.67, 0.91]
Postural control	SEBT composite score	<92.81	0.92	0.69	0.6	0.83 [0.72, 0.92]
Strength	Plantarflexion concentric	<2.24 Nm/kg	0.78	0.8	0.58	0.83 [0.72, 0.92]
Jump performance/Landing strategy	Drop jump contact time 20 cm	>0.23 s	0.86	0.78	0.64	0.87 [0.74, 0.96]

Abbreviations: AUC, area under the curve; CAIT, Cumberland Ankle Instability Tool; FAAM, foot and ankle ability measure; ROM, range of motion; SEBT, Star Excursion Balance Test.

A comprehensive correlation matrix was generated to examine associations among all parameters of the multidimensional assessment framework. CAIT and FAAM Sport demonstrated very strong intercorrelations, indicating substantial overlap in the constructs assessed. Furthermore, strong to very strong correlations were observed between the psychometric scores and functional outcomes (Table 8).

**Table 8:** Pearson's correlation coefficient (*r*) and *p* values for the recommended assessment framework, with Pearson's *r* indicating the correlation strength (moderate = very light blue and regular font; strong = blue and italic; very strong = dark blue and bold) and the *p* value assessing statistical significance.

	CAIT	FAAM Sport	ROM passive PF	ROM passive	External ankle rotation gait	SEBT Composite Score	Plantarflexion concentric	DJ contact time
CAIT								
FAAM Sport	***r*** = **0.905** ***p*** < **0.001**							
ROM passive PF	***r*** = **0.779** ***p*** < **0.001**	***r*** = **0.772** ***p*** < **0.001**						
ROM passive	***r*** = **0.768** ***p*** < **0.001**	***r*** = **0.819** ***p*** < **0.001**	***r*** = **0.945** ***p*** < **0.001**					
External ankle rotation gait	*r* = −0.427 *p* = 0.001	*r* = −0.423 *p* < 0.002	*r* = −0.496 *p* = 0.001	*r* = −0.441 *p* = 0.004				
SEBT Composite Score	*r* = *0.502* *p* < *0.001*	*r* = *0.595* *p* < *0.001*	*r* = *0.525* *p* < *0.001*	*r* = *0.591* *p* < *0.001*	*r* = −0.362 *p* = 0.007			
Plantar flexion conc.	*r* = *0.645* *p* < *0.001*	*r* = *0.622* *p* < *0.001*	*r* = *0.682* *p* < *0.001*	***r*** = **0.73** ***p*** < **0.001**	*r *= −0.217 *p* = 0.111	*r* = *0.551* *p* < *0.001*		
DJ contact time (20 cm)	***r*** = **−0.711** ***p*** < **0.001**	*r* = *−0.626* *p* < *0.001*	*r* = *−0.615* *p* < *0.001*	*r* = *−0.605* *p* < *0.001*	*r* = 0.278 *p* = 0.061	*r* = −0.486 *p* < 0.001	*r* = *−0.623* *p* < *0.001*	

Abbreviations: CAIT, Cumberland Ankle Instability Tool; DJ, drop jump; FAAM, foot and ankle ability measure; PF, plantarflexion; ROM, range of motion; SEBT, Star Excursion Balance Test.

## DISCUSSION

The most important finding of this study is sensitive parameters identifying functional impairments in patients with acute LAS within a robust study design. These specific parameters (CAIT, FAAM, SEBT Composite Score, concentric plantarflexion strength, passive plantarflexion and total ROM, transverse ankle kinematics during gait and DJ contact time) demonstrated large effect sizes and allowed the determination of clinically meaningful cut‐off values that may support structured functional assessment in the early phase after injury.

The functional impairments identified in the present study are largely consistent with previous evidence describing sensorimotor deficits during the early phase after acute LAS. Both the CAIT and FAAM have previously demonstrated substantial reductions in perceived ankle function within the first weeks after injury, supporting their role as sensitive patient‐reported outcome measures [10]. Similarly, impaired dynamic postural control, reflected by reduced SEBT performance, has repeatedly been reported following acute LAS and is likely influenced by the combined effects of reduced mobility, strength and neuromuscular control [10]. Strength deficits, particularly in PF, as well as reduced ankle ROM, have likewise been consistently described in previous studies during the early recovery phase [1, 27]. Furthermore, altered gait mechanics and modified landing strategies have been interpreted as protective movement adaptations aimed at reducing joint loading and maintaining ankle stability following injury [7, 17]. Collectively, these findings support the multidimensional nature of functional impairments after acute LAS and reinforce the rationale for selecting these parameters as the core components of the proposed assessment framework.

### Clinical integration

**Figure 2:**
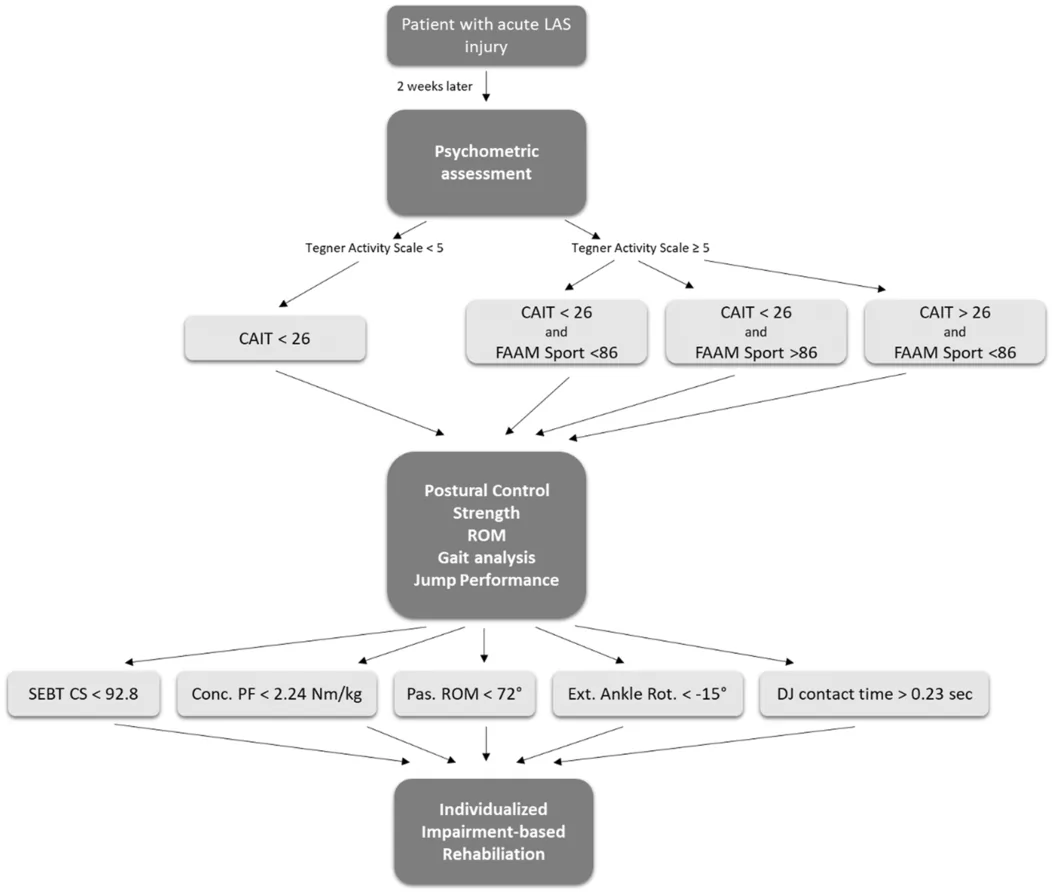
Clinical integration of the multidimensional assessment framework. CAIT, Cumberland Ankle Instability Tool; CS, Composite Score; DJ, drop jump; FAAM, Foot and Ankle Ability Measure; LAS, lateral ankle sprain; PF, plantarflexion; ROM, range of motion; SEBT, Star Excursion Balance Test.

Based on the observed effect sizes and inter‐domain associations (Table 8), a clinical integration of the multidimensional assessment framework to support clinicians in structured functional evaluation after acute LAS (Figure 2) was proposed. Due to its low testing burden and strong correlation with objective functional outcomes, psychometric assessment was established as the first screening step 2–3 weeks after injury. Strong correlations between patient‐reported outcome measures and functional test results suggest that self‐reported instability and functional limitations are likely related to underlying objective impairments rather than only subjective perception. Questionnaire scores may help identify individuals who warrant further functional assessment. However, the present findings do not support replacing objective physical testing with psychometric measures alone (Figure 2).

The observed associations suggest that psychometric measures may provide useful information regarding overall functional status. However, whether they can be used to reduce the need for physical testing requires prospective evaluation. Patients exceeding established cut‐off values can then be selectively assessed across the functional domains of postural control, strength, ROM, gait and jump performance. This staggered approach supports efficient allocation of clinical resources while preserving diagnostic precision and facilitating early, impairment‐based rehabilitation. The present assessment framework integrates objective, quantifiable measures across all domains, reducing examiner dependence and enabling standardized interpretation of functional deficits. Importantly, the use of defined test procedures and cut‐off–based decision criteria facilitates stratified clinical assessment rather than descriptive assessment alone.

The cut‐off values proposed in the present study should be interpreted as preliminary thresholds derived from an exploratory cross‐sectional analysis. As these thresholds were both derived and evaluated within the same study sample, their diagnostic performance may be overestimated. Consequently, external validation in independent cohorts and prospective studies is required before these cut‐off values can be recommended for routine clinical use.

Although the complete multidimensional assessment battery included several instrumented measures, not all of these assessments are required for routine clinical practice. Based on the present findings, a pragmatic core assessment may consist of the CAIT, FAAM Sport, the SEBT composite score and concentric PF strength. This combination requires only limited equipment (questionnaires, tape measure and, if available, a motorized dynamometer) and can be completed within a relatively short time. More advanced assessments, such as gait analysis using IMUs or jump analysis using force plates, may provide additional information but are primarily suited for specialized settings. Future studies should evaluate the feasibility and diagnostic performance of this simplified assessment strategy in routine clinical practice.

Our findings align with existing conceptual frameworks for acute LAS assessment while also providing meaningful extensions. Firstly, the ROAST recommendations of the International Ankle Consortium identify key domains relevant for evaluating acute LAS; however, these recommendations predominantly rely on subjective clinician rating or imprecise assessments [6]. While such approaches are pragmatic, their inherent subjectivity may limit sensitivity and reproducibility. Similarly, the PAASS framework provides a valuable consensus‐based approach for evaluating athletes following acute LAS by integrating pain, ankle impairments, athlete perception, sensorimotor control and sport/functional performance into return‐to‐sport decision‐making [29]. However, PAASS does not provide specific test recommendations or objective cut‐off values within these domains. In contrast, the present study identifies objective assessment tools and proposes preliminary cut‐off values for the early post‐injury phase. Furthermore, the recently proposed Ankle‐GO score represents a structured return‐to‐sport assessment that combines functional performance tests and patient‐reported outcomes to identify athletes who may be at increased risk of reinjury [25]. However, Ankle‐GO was developed for the later stages of rehabilitation and return‐to‐sport decision‐making rather than for the early assessment of acute impairments. Likewise, there is a notable conceptual overlap with the return‐to‐competition battery proposed by the German statutory accident insurance institution (Verwaltungs‐Berufsgenossenschaft, VBG) [3]. However, the clinical intent and timing approach differ substantially. Both the VBG battery and Ankle‐GO target athletes in the late rehabilitation phase, when impairments are expected to be stabilized and performance‐oriented benchmarks are appropriate. In contrast, the present study focuses on the acute post‐injury phase, characterized by variable symptom expression and evolving functional deficits. Our assessment framework therefore complements, rather than replaces, existing concepts.

## LIMITATIONS

This study is limited by its cross‐sectional design, which precludes conclusions regarding the prognostic value of the identified impairments or their direct association with the subsequent development of CAI. Furthermore, participants were recruited within the framework of a larger prospective study, and the sample size was not specifically determined for the present secondary analysis. Although several parameters demonstrated large effect sizes, the relatively modest sample size may limit the precision and external validity of the reported estimates and cut‐off values.

The focus on individuals with a first‐time LAS and no prior ankle injury, while enhancing internal validity, limits generalizability to populations with recurrent injuries or established instability where imaging is not routinely performed or recurrent injuries are common. Future longitudinal studies should therefore investigate the predictive value of the proposed assessment framework across more heterogeneous clinical cohorts.

Ankle ROM was assessed with the knee in an extended position. Although this standardized approach was applied consistently across groups, alternative protocols using a flexed knee may provide additional information by reducing the influence of the gastrocnemius on dorsiflexion measurements. Furthermore, the assessment of postural control required force plate technology, which may limit the transferability of this approach to routine clinical settings where such equipment is not readily available.

The exploratory nature of the present study required the evaluation of a large number of outcome parameters across multiple functional domains. Although no formal correction for multiple testing was applied, the interpretation of the findings was not based on *p* values alone but additionally considered effect sizes, clinical relevance and diagnostic performance. Nevertheless, the absence of multiplicity correction may have increased the risk of Type I error, and the reported findings should therefore be interpreted as exploratory. Future studies should validate the identified parameters in independent cohorts

The proposed multidimensional assessment framework includes several instrumented measures requiring specialized equipment, such as force plates, IMUs and dynamometry, which may not be readily available in routine clinical settings. Consequently, the feasibility of implementing the complete assessment battery may be limited outside specialized centres.

The present assessment was intentionally performed during the early post‐injury phase, when rehabilitation decisions are commonly initiated. However, functional impairments observed at this stage may reflect a combination of sensorimotor deficits, pain‐related adaptations, residual swelling, arthrogenic muscle inhibition and protective movement strategies. Due to the cross‐sectional design, the relative contribution of these mechanisms cannot be determined. Future longitudinal studies are required to investigate which deficits persist beyond the acute phase and whether they are associated with subsequent clinical outcomes.

Finally, all participants were assessed 2–3 weeks after injury and were able to complete the testing procedures. Therefore, the applicability of the proposed framework to patients with more severe symptoms or at earlier stages following injury remains uncertain.

## CONCLUSION

Individuals with acute LAS exhibit clinically relevant functional impairments across multiple domains (Psychometrics, Postural control, Strength, ROM, Gait/Running, Jump Performance/Landing Strategy) within the first weeks after injury. Several parameters showed strong discriminatory ability between injured and uninjured individuals and may represent promising candidates for structured multidimensional assessment in the early phase after acute LAS. Integrating patient‐reported outcomes with targeted functional testing may support structured clinical evaluation and help to identify impairment profiles that could be considered during rehabilitation planning following acute LAS. The proposed cut‐off values should be considered preliminary and require validation in independent cohorts before routine clinical implementation. Future longitudinal studies are needed to determine their prognostic relevance and clinical utility.

## AUTHOR CONTRIBUTIONS

Janina Tennler, Christian Raeder, Arthur Praetorius and Christian Schoepp contributed to the conceptualization and study design. Janina Tennler was responsible for the data collection, data analysis and drafting of the manuscript. Christian Raeder, Arthur Praetorius, Sebastian Colcuc, Eva S. Steinhausen, Marcel Dudda and Christian Schoepp contributed to revising the manuscript. All authors read and approved the final version of the manuscript.

## FUNDING

Open Access Publication Fund of the University of Duisburg‐Essen; DGUV (German Social Accident Insurance), Grant/Award Number: FF‐FR 0329.

## CONFLICT OF INTEREST STATEMENT

The authors declare no conflicts of interest.

## ETHICS STATEMENT

This study was approved by Ärztekammer Nordrhein (ref: 2021236 28/09/2021). All participants provided written informed consent prior to participation in the study.

## Supporting information

Supporting File 1:

## Data Availability

Pseudonymized raw data of all assessed test domains, as well as relevant demographic variables, are available upon reasonable request from the corresponding author.
