# Supplementary material for: Early functional deficits following first‐time acute lateral ankle sprain: A multidimensional assessment framework with clinically relevant cut‐off values
Source: J Exp Orthop. 2026 Aug 27;13(3):e70892. doi: 10.1002/jeo2.70892 (PMC13521570; doi:10.1002/jeo2.70892)
Supplement: Supplementary file 1 [file JEO2-13-e70892-s001.docx]

**Appendix**

**TAB. 9:** Comparison of group means (mean ± SD) for time-to-boundary parameters. Significance was determined using an unpaired t-test (†) or Mann-Whitney U test (‡). Effect sizes were calculated using Cohen’s *d*. Significance levels: *p<.05, **p<.01, ***p<.001; mediolateral (ML), anterior-posterior (AP)

|  | | **Injured n=36** | **Uninjured n=36** | ***P* Value** | **Cohen’s *d*** |
| --- | --- | --- | --- | --- | --- |
|  | | **(not feasible n=1)** | **(not feasible n=1)** |  |  |
| **Eyes open** | **ML min** | 0.65 ± 0.19 | 0.73 ± 0.13 | 0.166† | -0.335 |
|  | **AP min** | 1.41 ± 0.61 | 1.85 ± 0.69 | **0.006**†** | -0.672 |
|  | **min** | 0.64 ± 0.18 | 0.73 ± 0.26 | 0.121† | -0.375 |
|  | **ML min mean** | 2.76 ± 0.53 | 2.87 ± 0.69 | 0.502† | -0.161 |
|  | **AP min mean** | 4.46 ± 0.94 | 5.12 ± 0.72 | **0.019*†** | -0.573 |
|  | **Min mean** | 2.5 ± 0.56 | 2.73 ± 0.75 | 0.149† | -0.349 |
|  | **ML min SD** | 1.98 ± 0.32 | 1.99 ± 0.39 | 0.882† | -0.036 |
|  | **AP min SD** | 2.19 ± 0.25 | 2.13 ± 0.21 | 0.301† | 0.249 |
|  | **Min SD** | 1.75 ± 0.36 | 1.86 ± 0.42 | 0.231† | -0.289 |
|  | | **(not feasible n=6)** | **(not feasible n=1)** |  |  |
| **Eyes closed** | **ML min** | 0.3 ± 0.1 | 0.31 ± 0.08 | 0.483† | -0.176 |
|  | **AP min** | 0.7 ± 0.33 | 0.75 ± 0.34 | 0.531† | -0.157 |
|  | **min** | 0.29 ± 0.09 | 0.31 ± 0.09 | 0.378† | -0.221 |
|  | **ML min mean** | 1.63 ± 0.44 | 1.82 ± 0.46 | 0.080† | -0.442 |
|  | **AP min mean** | 2.91 ± 0.68 | 3.28 ± 0.64 | **0.026*†** | -0.567 |
|  | **Min mean** | 1.23 ± 0.38 | 1.33 ± 0.38 | 0.256† | -0.285 |
|  | **ML min SD** | 1.66 ± 0.45 | 1.87 ± 0.39 | 0.052† | -0.493 |
|  | **AP min SD** | 1.99 ± 0.27 | 2.08 ± 0.28 | 0.192† | -0.328 |
|  | **Min SD** | 1.19 ± 0.45 | 1.24 ± 0.42 | 0.67† | -0.107 |

**TAB. 10:** Comparison of group means (mean ± SD) for Star Excursion Balance Test parameters. Significance was determined using an unpaired t-test (†) or Mann-Whitney U test (‡). Effect sizes were calculated using Cohen’s *d*. Significance levels: *p<.05, **p<.01, ***p<.001

|  | **Injured (n=36)** | **Uninjured(n=36)** | ***P* Value** | **Cohen’s *d*** |
| --- | --- | --- | --- | --- |
| **Anterior [cm]** | 55 ± 8 | 64 ± 11 | **<.001***†** | -0.987 |
| **Posteromedial [cm]** | 102 ± 11 | 116 ± 13 | **<.001***†** | -1.149 |
| **Posterolateral [cm]** | 98 ± 15 | 112 ± 14 | **<.001***†** | -1.011 |
| **Composite Score** | 89 ± 11 | 102 ± 10 | **<.001***†** | -1.223 |

**TAB. 11:** Comparison of group means (mean ± SD) for joint repositioning sense parameters. Significance was determined using an unpaired t-test (†) or Mann-Whitney U test (‡). Effect sizes were calculated using Cohen’s *d*. Significance levels: *p<.05, **p<.01, ***p<.001

|  | **Injured n=36** | **Uninjured n=36** | ***P* Value** | **Cohen’s *d*** |
| --- | --- | --- | --- | --- |
| **Constant error** | 0.1 ± 2.1 | 0.4 ± 1.6 | 0.606† | -0.122 |
| **Variable error** | 2.1 ± 1.1 | 2.1 ± 1.1 | 0.928† | -0.021 |
| **Absolute error** | 2.3 ± 1.3 | 1.9 ± 1.1 | 0.149† | 0.344 |

**TAB. 12:** Comparison of group means (mean ± SD) for drop jump parameters. Significance was determined using an unpaired t-test (†) or Mann-Whitney U test (‡). Effect sizes were calculated using Cohen’s *d*. Significance levels: *p<.05, **p<.01, ***p<.001; reactive strength index (RSI), vertical ground reaction force (VGRF)

|  | **Injured n=36**  **(not feasible n=13)** | **Uninjured n=36** | ***P* Value** | **Cohen’s *d*** |
| --- | --- | --- | --- | --- |
| **Max jump height [cm]** | 14.0 ± 7.1 | 22.6 ± 6.5 | **<.001***‡** | -0.652 |
| **Min contact time [s]** | 0.27 ± 0.06 | 0.2 ± 0.04 | **<.001***†** | 1.619 |
| **Max RSI** | 0.5 ± 0.37 | 1.05 ± 0.47 | **<.001***‡** | -0.678 |
| **Max rel. peak VGRF [N/kg]** | 46.6 ± 9.8 | 59.9 ± 11.3 | **<.001***†** | -1.242 |
| **Max rel. peak VGRF of involved leg [N/kg]** | 21.0 ± 6.8 | 30.5 ± 7.0 | **<.001***†** | -1.368 |

**FIG. 3:** a) Mean trajectories of **vertical ground reaction force normalized to bodyweight** (y-axis) during gait (x-axis: gait cycle 0-100%) for the injured group (black n=33) and uninjured group (red n=36). b) The unpaired samples t-test statistic SPM(t).

**FIG. 4:** a) Mean trajectories of **sagittal plane hip, knee and ankle kinematics** (y-axis: joint angle (°)) during gait (x-axis: gait cycle 0-100%) for the injured group (black, hip n=32, knee n=32, ankle n=32) and uninjured group (red, hip n=35, knee n=36, ankle n=27). b) The unpaired samples t-test statistic SPM(t).

**FIG. 5:** a) Mean trajectories of **frontal plane hip, knee and ankle kinematics** (y-axis: joint angle (°)) during gait (x-axis: gait cycle 0-100%) for the injured group (black, hip n=31, knee n=32, ankle n=30) and uninjured group (red, hip n=36, knee n=36, ankle n=26). b) The unpaired samples t-test statistic SPM(t).

**FIG. 6:** a) Mean trajectories of **transverse plane hip, knee and ankle kinematics** (y-axis: joint angle (°)) during gait (x-axis: gait cycle 0-100%) for the injured group (black, hip n=32, knee n=30, ankle n=28) and uninjured group (red, hip n=36, knee n=36, ankle n=27). b) The unpaired samples t-test statistic SPM(t).

**FIG. 7:** a) Mean trajectories of **vertical ground reaction force normalized to bodyweight** (y-axis) during running (x-axis: running cycle 0-100%) for the injured group (black n=19) and uninjured group (red n=36). b) The unpaired samples t-test statistic SPM(t).

**FIG. 8:** a) Mean trajectories of **sagittal plane hip, knee and ankle kinematics** (y-axis: joint angle (°)) during running (x-axis: running cycle 0-100%) for the injured group (black, hip n=19, knee n=19, ankle n=18) and uninjured group (red, hip n=36, knee n=36, ankle n=27). b) The unpaired samples t-test statistic SPM(t).

**FIG. 9**: a) Mean trajectories of **frontal plane hip, knee and ankle kinematics** (y-axis: joint angle (°)) during running (x-axis: running cycle 0-100%) for the injured group (black, hip n=19, knee n=19, ankle n=15) and uninjured group (red, hip n=35, knee n=36, ankle n=25). b) The unpaired samples t-test statistic SPM(t).

**FIG. 10:** a) Mean trajectories of **transverse plane hip, knee and ankle kinematics** (y-axis: joint angle (°)) during running (x-axis: running cycle 0-100%) for the injured group (black, hip n=19, knee n=19, ankle n=15) and uninjured group (red, hip n=36, knee n=36, ankle n=25). b) The unpaired samples t-test statistic SPM(t).

**FIG. 11:** a) Mean trajectories of vertical ground reaction force normalized to bodyweight (y-axis) during the single-leg drop landing (x-axis: 200ms pre/post initial contact) for the injured group (black n=27) and uninjured group (red n=32). b) The unpaired samples t-test statistic SPM(t).

**FIG. 12:** a) Mean trajectories of **sagittal plane hip, knee and ankle kinematics** (y-axis: joint angle (°)) during the single-leg drop landing (x-axis: 200ms pre/post initial contact) for the injured group (black, hip n=31, knee n=31, ankle n=29) and uninjured group (red, hip n=35, knee n=35, ankle n=25). b) The unpaired samples t-test statistic SPM(t).

**FIG. 13:** a) Mean trajectories of **frontal plane hip, knee and ankle kinematics** (y-axis: joint angle (°)) during the single-leg drop landing (x-axis: 200ms pre/post initial contact) for the injured group (black, hip n=31, knee n=31, ankle n=29) and uninjured group (red, hip n=35, knee n=34, ankle n=25). b) The unpaired samples t-test statistic SPM(t).

**FIG. 14:** a) Mean trajectories of **transverse plane hip, knee and ankle kinematics** (y-axis: joint angle (°)) during the single-leg drop landing (x-axis: 200ms pre/post initial contact) for the injured group (black, hip n=31, knee n=31, ankle n=29) and uninjured group (red, hip n=35, knee n=35, ankle n=26). b) The unpaired samples t-test statistic SPM(t).
